# Genetic Markers as Risk Factors for the Development of Impulsive-Compulsive Behaviors in Patients with Parkinson’s Disease Receiving Dopaminergic Therapy

**DOI:** 10.3390/jpm11121321

**Published:** 2021-12-07

**Authors:** Anna Fedosova, Nataliya Titova, Zarema Kokaeva, Natalia Shipilova, Elena Katunina, Eugene Klimov

**Affiliations:** 1 Lomonosov Moscow State University, Faculty of Biology, Leninskie Gory, 1, Building 12, 119234 Moscow, Russia; annetfedosova@yandex.ru (A.F.); zaremak@inbox.ru (Z.K.); 9395001@mail.ru (E.K.); 2 Pirogov Russian National Research Medical University, Department of Neurology, Neurosurgery and Medical Genetics, Ostrovitianova, 1, 117997 Moscow, Russia; natali.33@mail.ru (N.S.); elkatunina@mail.ru (E.K.); 3 Federal State Budgetary Institution “Federal Center of Brain Research and Neurotechnologies” of the Federal Medical Biological Agency, Department of Neurodegenerative Disorders, Ostrovitianova, 1, Building 10, 117997 Moscow, Russia

**Keywords:** Parkinson’s disease (PD), impulsive-compulsive disorders (ICD), dopaminergic therapy, genetic markers, pharmacogenetic, polymorphisms

## Abstract

Impulsive–compulsive and related behavioral disorders (ICD) are drug-induced non-motor symptoms of Parkinson’s disease (PD). Recently research has focused on evaluating whether ICD could be predicted and managed using a pharmacogenetic approach based on dopaminergic therapies, which are the main risk factors. The aim of our study was to evaluate the role of candidate genes such as *DBH*, *DRD2*, *MAOA*, *BDNF*, *COMT*, *SLC6A4*, *SLC6A3*, *ACE*, *DRD1* gene polymorphisms in the pathogenesis of ICD in PD. We compared patients with PD and ICD (*n* = 49), patients with PD without ICD (*n* = 36) and a healthy control group (*n* = 365). ICD was diagnosed using the QUIP questionnaires and specific diagnostic criteria for subtypes of ICD. Genotyping was conducted using a number of PCR techniques and SNaPshot. Statistical analysis was performed using WinPepi and APSampler v3.6 software. PCA testing was conducted using RStudio software v1.4.1106-5. The following substitutions showed statistically significant correlations with PD and ICD: *DBH* (rs2097629, rs1611115), *DRD2* (rs6275, rs12364283, rs1076560), *ACE* (rs4646994), *DRD1* (rs686), *BDNF* (rs6265), these associations are novel in Russian PD patients. Our findings suggest that polymorphisms in *DBH*, *BDNF*, *DRD2*, *ACE* genes in Russian subjects are associated with an increased risk of ICD development.

## 1. Introduction

Parkinson’s disease (PD) is a syndromic condition and is phenotypically associated with a range of motor and nonmotor symptoms (NMS) [1]. Various types of disease-related and drug-induced NMS are recognized and impulsive-compulsive disorders (ICD) that include hypersexuality, compulsive overeating, compulsive shopping, pathological gambling, punding, hobbyism and dopamine dysregulation syndrome are challenging dopaminergic therapy related NMS of key clinical significance [2,3,4,5]. The subtle and initial symptoms of ICD are often overlooked in clinical practice, since they are quite difficult to recognize at early stages. Early recognition is important as studies suggest that ICD related abnormal behaviors significantly worsen the parameters of daily activities and quality of life of patients with PD worsening psychological stress, depression, anxiety and sleep disorders. Unrecognized and untreated, these disorders can lead to devastating consequences, including financial collapse and bankrupcy, divorce, dismissal from work, disruption of social activities, unsanitary living conditions and somatic complications. The estimated frequency of ICD in PD patients varies greatly in different studies—from 3.5% to 42.8% [3,6,7] due to the use of different study designs, questionnaires, scales, as well as different cultural, social, ethnic and economic characteristics of the patients. There is a clear association between the use of dopaminergic therapy (especially dopamine receptor agonists) and ICD development. Other risk factors for the development of ICD include male gender, young age, early PD development, history of ICD, substance and alcohol abuse, bipolar disorder, depression, smoking, and being unmarried [3,8,9,10,11,12].

Genetic factors are thought to play a certain role in the development of ICD. The involved genes are those encoding receptors or transporters involved in dopamine metabolism, or genes that regulate the activity of enzymes involved in the breakdown pathways of the main neurotransmitters, i.e., dopamine, serotonin, norepinephrine, glutamate [2,13,14,15,16,17,18,19,20]. As an example, addictive behavior in early PD has been linked to *DRD3* variant [18].

Our central hypothesis is based on other research addressing genetic risk factors for ICD using candidate genetic panel-based predictability of ICD in PD and most suggest that related gene products with ICD link are involved in the dopamine metabolizing pathways.

We hypothesized that some proposed ICD markers could be used as a pre-diagnostic marker prior to overt clinical manifestations of the disease. These data could then help to manage and personalize therapy at early stages of PD when there is minimal neuronal degradation. 

The study was aimed at evaluating the role of *DBH*, *DRD2*, *MAOA*, *BDNF*, *COMT*, *SLC6A4*, *SLC6A3*, *ACE*, *DRD1* gene polymorphisms in the development of ICD in PD patients receiving dopaminergic therapy. To the best of our knowledge, it was the first genetic study evaluating ICD in Russian PD patients.

## 2. Materials and Methods

### 2.1. Patients

The 386 PD patients were examined over the period from 2015 to 2018. PD diagnosis was made based on the UK Parkinson’s Disease Society Brain Bank clinical diagnostic criteria [21]. The inclusion and exclusion criteria were used for patient enrolment to the study. The inclusion criteria were as follows: age over 40 years, the use of dopaminergic therapy, the patient’s informed written consent to participate in the study. For the control group, the inclusion criterion was the history of treatment with dopamine receptor agonists (DA) for at least 3 years. The exclusion criteria were as follows: dementia of any grade (based on the DSM-IV criteria [American Psychiatric Association, 2000], MMSE total score < 24).

The screening survey for the detection of ICD in PD patients was conducted using QUIP-Short and QUIP-Full questionnaires [22,23]. 

These questionnaires revealed ICD related symptoms in 78 (20.2%) subjects. Subsequently, specific diagnostic criteria were applied to confirm each subtype of ICD. Pathological gambling and compulsive overeating were confirmed based on the DSM-IV diagnostic criteria; compulsive shopping—based on the criteria developed by S. McElroy et al. [24]; hypersexuality—based on the criteria developed by V. Voon et al. [25]; punding and hobbyism—based on the criteria developed by A. Evans et al. [26], dopamine dysregulation syndrome—based on the criteria developed by G. Giovannoni et al. [27]. Thus, the main group included patients who had been found to have ICD based on the QUIP screening survey and the use of comprehensive diagnostic criteria (*n* = 49; PD + ICD; PD1). The control group included 36 PD patients who did not demonstrate abnormal behaviors or ICD (PD2). Demographic and clinical data of the patients are shown in Table 1. The population sample in this study is ethnically homogeneous and represent a white Caucasian population.

### 2.2. Ethical Principles

The study was conducted in accordance with the requirements of the World Medical Association (WMA)’s Declaration of Helsinki. All patients gave their written informed consent to participate in the study. 

### 2.3. Methods

Laboratory tests included collection of venous blood samples in PD patients of the main group (*n* = 49; PD + ICD) and the control group (*n* = 36). Blood samples were stored in vacuum tubes with EDTA K2/K3 specially designed for laboratory whole blood studies. EDTA fillers (ethylenediaminacetic acid) bind calcium ions, creating stable complexes and was used as an anticoagulant in this study. Blood sampling was performed at N.I. Pirogov Municipal Clinical Hospital No.1 (N.I. Pirogov Russian National Research Medical University) and a consultative medical office for patients with extrapyramidal symptoms of catchment of the District Neurology Department of Central Administrative District of Moscow City. 

Population control group blood samples (*n* = 365; control) were provided by blood transfusion station on condition of anonymity. The population control aim is needed to provide a natural baseline for mutation frequencies. The estimated number of PD patients in Russia is approximately 210,000 people (prevalence of 30–140/100,000) and thus it is necessary to provide control data as far as possible so as to account for natural variations. We used a fully health screened blood donor group where all the donors had passed a rigorous medical examination with exclusion of those with family history of neurodegenerative disorders, PD, dementia as well as any behavioral or mental health issues.

Genotype frequencies of selected gene substitutions were estimated:

- *ACE* (rs4646994)
- *BDNF* (rs2049046, rs6265)
- *COMT* (rs4680)
- *DBH* (rs141116007, rs2097629, rs1611115)
- *DRD1* (rs686)
- *DRD2* (rs1799732, rs6275, rs2283265, rs12364283, rs1076560)
- *MAOA* (VNTR)
- *SLC6A3* (rs27072)
- *SLC6A4* (rs38130034)

#### 2.3.1. DNA Isolation

DNA was isolated from whole blood samples using columns according to the manufacturer’s instructions (IG Spin DNA Prep 100 kit, manufactured by Isogen Laboratory LLC, Russia).

#### 2.3.2. PCR Testing

The allelic analysis was conducted using polymerase chain reaction (PCR)-based techniques: PCR, PCR-RFLP (the combination of the polymerase chain reaction with the restriction fragment length polymorphism analysis), real-time PCR, and SNaPshot (single nucleotide polymorphism genotyping using allele-specific PCR and fluorescence melting curves) [28]. The sequences of primers (manufactured by DNA-Synthesis LLC, Moscow, Russia) are shown in Supplementary Material Tables S1 and S2. 

PCR testing was carried out using HS Taq DNA polymerase and ScreenMix-HS test kits (manufactured by Evrogen, Moscow, Russia), and the T100 device (Bio-Rad Laboratories, Inc, Hercules, CA, USA). The following PCR cycling parameters were used: 94 °C–3 min; 40–45 cycles: 94 °C—20 s, To °C—15 s, 72 °C—30 s; 72 °C—5 min, where To is the primer annealing temperature (see Supplementary Material Tables S1 and S2). 

Real-time PCR was conducted using qPCRmix-HS and qPCRmix-HS SYBR test kits (manufactured by Evrogen, Moscow, Russia) and the StepOnePlus Real Time PCR System device (Applied Biosystems, Waltham, MA, USA). The fluorescence detection was performed at FAM/VIC channels.

#### 2.3.3. Restriction Analysis

The restriction analysis of PCR products was conducted in the conditions described by the restriction endonuclease manufacturer (SibEnzyme Ltd., Novosibirsk, Russia). The table describing restriction endonucleases used and DNA fragments obtained is presented in Supplementary Material Table S3.

### 2.4. Statistical Analysis

A two-tailed Fisher exact test (Fi) was used to reliably compare small samples during the assessment of gene substitution association. The calculations were performed using WinPepi software, v.11.65 (http://www.brixtonhealth.com/pepi4windows.html) (accessed on 23 August 2016) [29]. The results with Fisher’s *p*-value < 0.05 were considered statistically significant. The mode of inheritance (dominant or recessive) was determined in accordance with the Akaike information criterion. 

The groups of PD patients with ICD symptoms while on dopaminergic therapy, PD patients not experiencing impulse control disorders, and the population control group were used for comparative analysis.

The following groups were compared: PD1 versus PD2, PD1 versus control, PD2 versus control, PD1 + PD2 versus control.

The detection of complex genotypes associated with a trait was conducted using APSampler v3.6 [30] polygenic data analysis software based on common statistical tests (Fisher’s exact test, Bonferroni adjustment for *p*-value and FDR) as well as the permutation test algorithm, which allowed to analyze associations in small samples.

### 2.5. Principal Component Analysis

PCA was applied to ensure best visualization of differences in a data set with many variables. The data set is adjusted to the new coordinate system in such a way that the most significant variance is detected at the first coordinate, and each subsequent coordinate is orthogonal to the last one and has a smaller variance. Thus, a set of X correlated variables for Y samples is transformed into a set of *p* uncorrelated principal components for the same samples. The analysis was conducted using RStudio software.

## 3. Results

### 3.1. Association between the Genetic Markers in PD Patients without ICD (PD2 Group)

The association between PD without ICD and patient genotypes was evaluated by statistical analysis using the WinPepi software. The mode of inheritance was determined using the Akaike information criterion. The mode with the lowest *p*-value according to the Fisher’s test was considered the correct one. All data obtained for SNP genes evaluated are shown in Table 2.

Our study demonstrated statistically significant results for several substitutions (Table 2):

- rs2097629 substitution in the *DBH* gene (9q34.2, 1434 + 1579A > G, 3′ region) is associated with the disease. Analysis of allele frequencies of this substitution also showed an association of the G allele with PD (*p* = 0.016, OR = 2.97, CI95% [1.17–8.97]). The mode of inheritance was found to be dominant.
- rs1611115 substitution in the *DBH* gene (9q34.2, 1021T > C, 5′ region) is associated with the disease. Analysis of the frequencies of alleles of this substitution also showed an association of the allele with PD (*p* = 2.8 × 10^−3^, OR = 3.71, CI95% [1.46–8.77]). The mode of inheritance was found to be recessive.
- rs6265 substitution in the *BDNF* gene (11p14.1, 196G > A, Val66Met, Exon 2) it is associated with the disease. Analysis of allele frequencies of this substitution also showed an association of Allele A with PD (*p* = 6.7 × 10^−3^, OR = 2.83, CI95% [1.27–6.29]). The mode of inheritance was found to be dominant.
- rs6275 substitution in the *DRD2* gene (11q23.2, 939T > C, His313His, Exon 7) is associated with the disease. Analysis of allele frequencies of this substitution also showed an association of the T allele with PD (*p* = 2.9 × 10^−8^, OR = 9.00, CI95% [3.97–20.14]). The mode of inheritance was found to be recessive.
- rs12364283 substitution in the *DRD2* gene (11q23.2, 4047A > G, 5′ region) is associated with the disease. Analysis of allele frequencies of this substitution also showed an association of the T allele with PD (*p* = 0.012, OR = 3.30, CI95% [1.25–10.19]). The mode of inheritance was found to be recessive.
- rs686 substitution in the *DRD1* gene (5q35.1, 7464G > A, 3′ region) is associated with the disease. Analysis of allele frequencies of this substitution also showed an association of Allele A with PD (*p* = 0.017, OR = ∞, CI95% [1.3583–∞]). The mode of inheritance was found to be dominant.

A polygenic analysis was conducted to evaluate the predisposition to PD in the group of patients versus the population control group. The analysis was carried out based on the genotypes of 36 PD patients and 365 residents of Moscow and the Moscow region (population control group) assessed for six polymorphic sites of four candidate genes. The results of the polygenic analysis are shown in Table 3 and Table 4. Combinations of genotypes or individual genotypes and alleles were considered statistically significant if the *p*-value (Westfall–Young) was <0.001.

A total of four complex genotypes were found to meet our parameters (OR > 1). In three of four cases, the rs6275 TT substitution genotype was found in the *DRD2* gene, which resulted in about seven-fold increase in the risk of PD development (Table 3). 

Two protective variants were determined during the complex genotype analysis. In both cases, the *DRD2* rs6275:C allele is present, which is associated with about seven-fold decreased risk of PD development (Table 4).

### 3.2. Association between the Genetic Markers and ICD in PD Patients (PD1 Group)

The association between PD patient genotypes and ICD development was evaluated by statistical analysis that included comparison of genotypes in the following groups: PD + ICD versus control group and PD + ICD versus PD without ICD group (used as a control group in this case). The mode of inheritance was determined using the Akaike information criterion. The mode with the lowest *p*-value according to the Fisher’s test was considered the correct one. All data obtained for SNP genes evaluated are shown in Table 5.

Our study demonstrated statistically significant results for several substitutions (Table 5):

- rs1611115 substitution in the *DBH* gene (9q34.2, 1021T > C, 5′ region) is associated with the disease. Analysis of the frequencies of alleles of this substitution also showed an association of the allele with PD (*p* = 2.8 × 10^−3^, OR = 3.71, CI95% [1.46–8.77]). The mode of inheritance was found to be dominant.
- rs6265 substitution in the *BDNF* gene (11p14.1, 196G > A, Val66Met, Exon 2) it is associated with the disease. Analysis of allele frequencies of this substitution also showed an association of Allele A with PD (*p* = 6.7 × 10^−3^, OR = 2.83, CI95% [1.27–6.29]). The mode of inheritance was found to be dominant.
- rs6275 substitution in the *DRD2* gene (11q23.2, 939T > C, His313His, Exon 7) is associated with the disease. Analysis of allele frequencies of this substitution also showed an association of the T allele with PD (*p* = 2.9 × 10^−8^, OR = 9.00, CI95% [3.97–20.14]). The mode of inheritance was found to be dominant.
- rs12364283 substitution in the *DRD2* gene (11q23.2, 4047A > G, 5′ region) is associated with the disease. Analysis of allele frequencies of this substitution also showed an association of the T allele with PD (*p* = 0.012, OR = 3.30, CI95% [1.25–10.19]). The mode of inheritance was found to be recessive.
- rs1076560 substitution in the *DRD2* gene (11q23.2, 67314C > A, Intron 6) is associated with the disease. Analysis of allele frequencies of this substitution also showed an association of the T allele with PD (*p* = 0.012, OR = 3.30, CI95% [1.25–10.19]). The mode of inheritance was found to be dominant.
- rs4646994 substitution in *ACE* gene (11q23.2, I/D 289bp, Intron 16) is associated with the disease. Analysis of allele frequencies of this substitution also showed an association of the T allele with PD (*p* = 0.024, OR = 2.64, CI95% [1.12–7.22]). The mode of inheritance was found to be dominant.

A polygenic analysis was conducted to evaluate the predisposition to ICD in the group of patients versus the population control group. The analysis was carried out based on the genotypes of 45 PD patients and 365 residents of Moscow and the Moscow region (population control group) assessed for six polymorphic sites of four candidate genes. The results of the polygenic analysis are shown in Table 6 and Table 7. Combinations of genotypes or individual genotypes and alleles were considered statistically significant if the *p*-value (Westfall–Young) was <0.001.

A total of four complex genotypes were found to be associated with ICD (OR > 1). In three of four cases, there is a *BDNF*_rs6265:A allele, which makes a significant contribution to the development of ICD in PD patients receiving long-term dopaminergic therapy (Table 6).

Two protective variants were determined during the complex genotype analysis. In both cases, a *BDNF*_rs6265:G allele is present (Table 7).

Only the following genotype combinations were found to be statistically significant in the analysis of PD1 versus PD2 groups: CT + CC, rs6275 in the *DRD2* gene (11q23, 939T > C, His313His, Exon 7). The analysis of prevalence of this substitution demonstrated a correlation between the C allele with PD + ICD (*p* = 0.026, OR = 2.85, CI95% [1.04–7.81]). The mode of inheritance was found to be dominant.

No additional statistical analysis was conducted in respect of a single *DRD2* gene when comparing PD + ICD (PD1) versus PD without ICD (PD2, control).

### 3.3. Principal Component Analysis

Principal component analysis (PCA) was conducted using R-Studio software based on genotype data in 49 patients of the PD + ICD group, 36 PD patients without ICD and 201 patients from the population control group. The following substitutions demonstrating statistically significant correlation with the disease development were selected for the analysis: *DBH* (rs2097629, rs1611115), *DRD2* (rs6275, rs12364283, rs1076560), *ACE* (rs4646994), *DRD1* (rs686), *BDNF* (rs6265).

PCA allowed to identify three statistically significant clusters that corresponded to the baseline data.

**Figure 1:**
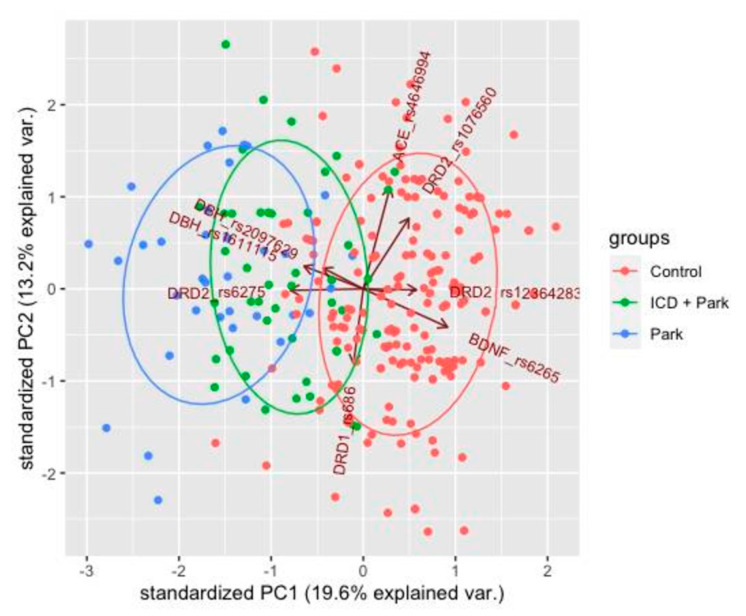
PCA results. PC1, PC2 are the principal components that explain 19.6% and 13.2% of the variance, i.e., the percentages of the total spread in points that falls on each of the new coordinates. Each sample has its own coordinates on the multidimensional plane. These coordinates consist of all possible vectors of the effects of DBH substitutions *DBH* (rs2097629, rs1611115), *DRD2* (rs6275, rs12364283, rs1076560), *ACE* (rs4646994), *DRD1* (rs686), *BDNF* (rs6265). In the obtained coordinate system, the samples are distributed into three clusters corresponding to the original data groups. DBH, *DRD2, BDNF* gene substitutions demonstrate the greatest impacts on the distribution of control, PD1 and PD + ICD (PD2) groups. The heterogeneity of the PD groups (PD1 + PD2) was due to the diverse effects of *DRD2* gene substitutions on the disease development.

The greatest differences in the groups of PD patients and the control group were observed in respect of *DBH*, *DRD2*, *BDNF* gene substitutions. The heterogeneity of the PD group was due to the diverse effects of *DRD2* gene substitutions on the disease development (Figure 1).

These findings are supported by the analysis of associations between the genetic markers and ICD in PD patients.

## 4. Discussion

Our study reports the key findings that variants rs1611115 *DBH*, rs6265 *BDNF*, rs6275 *DRD2* rs12364283 *DRD2*, rs1076560 *DRD2*, rs4646994 *ACE* are associated with an increased ICD risk among PD patients. To the best of our knowledge, we believe that this is the first report of clinical genetic testing conducted in patients with PD and ICD in Russia. We will now discuss individual aspects of these findings. 

### 4.1. Association between the Genetic Markers and PD

A range of genetic markers have been associated with behavioral and other drug induced nonmotor issues in PD. For instance, the *DRD2* rs1799732 and DRD3 rs6280 gene polymorphisms have been linked to levodopa induced gastrointestinal symptoms [19]. Post-traumatic stress disorder as well as sleep dysfunction arising from chronic stress have also been linked to SNP *DRD2* density and *DRD2* gene polymorphisms [31,32]. In PD, ICD is widely regarded as a drug induced behavioural issue and we now discuss relevant and related genetic basis. 

The *DBH* gene encodes a protein of the same name that is responsible for the conversion of dopamine to norepinephrine. The *DBH* gene sequence includes a coding DBH antisense RNA 1—DBH-AS1 region; this non-coding protein transcript may regulate the *DBH* gene translation. The dominant G allele of the rs2097629 substitution was shown to be associated with the PD development (*p* = 0.016) with OR = 2.97, 95% CI [1.17–8.97]). This substitution located in 3′ region of the gene has been postulated to produce a negative effect on dopamine metabolism by reducing the dopamine beta-hydroxylase synthesis [33]. The 5′ region of the gene includes a rs1611115 substitution [31], the recessive Allele C of which is also implicated in the pathogenesis of PD (*p* = 2.8 × 10^−3^) with OR = 3.71, 95% CI [1.46–8.77]. This substitution significantly regulates the enzyme plasma activity [34]. In this regard, the impaired function of the dopaminergic system increases the risk of PD development. 

We also interrogated the *BDNF* gene which encodes a protein that is active in the spinal cord and the brain and regulates the growth, differentiation and functioning of neurons. The dominant Allele A of the rs6265 substitution increases the risk of PD development (*p* = 6.7 × 10^−3^), with OR = 2.83, 95% CI [1.27–6.29]. This substitution is located in Exon 2 of the *BDNF* gene and leads to the Val66Met amino acid substitution. The Met allele is associated with abnormal intracellular packaging of the BDNF precursor and a decrease in the cell production of mature BDNF [35]. The rs6265 substitution is also associated with obsessive-compulsive disorder (OCD), attention-deficit/hyperactivity disorder, anxiety disorders and could be operative via functional alterations within the hippocampus and prefrontal cortex [36]. Moreover, this substitution is also associated with the development of Alzheimer’s disease as it causes progressive memory loss and cognitive impairment [37].

The *DRD2* gene encodes the dopamine receptor, which is a G-coupled protein located on the surface of neurons and inhibiting dopamine-induced adenylate cyclase activity [32]. TT genotype of the rs6275 substitution increases the risk of PD development (*p* = 2.9 × 10^−8^) with OR = 9.00, 95% CI [3.97–20.14]. The C allele is dominant, and the T allele is recessive. This substitution is located in Exon 7 of the dopamine D2 receptor encoding gene and the T allele affects the stability of the DRD2 transcript and its translation efficiency [38]. The major effect is expected on the presynaptic membrane, where the D2 dopamine receptor activates the dopamine reuptake. With a decrease in the amount of DRD2 on the presynaptic membrane, dopamine accumulation in the synaptic cleft should be expected. This may result in excessive activation of the downstream dopamine receptors and an increased response on the dopamine release. The TT genotype is likely to result in a decreased reuptake from the synapse due to imbalance of the number of D2 dopamine receptors and dopamine, which can lead to striatal dopamine depletion. The 5′ region of the gene includes a rs12364283 substitution, the recessive Allele A of which is associated with the PD development (*p* = 0.012) with OR = 3.30, 95% CI [1.25–10.19]. This substitution has been found to be associated with behavioral disorders and possibly also with pathogenesis of PD [39] D1 receptor gene (*DRD1*) is located at 5q35.1 and has two exons. DRD1 is one of the most common dopaminergic receptors in the central nervous system. This gene is involved in social cognition, attention, reinforcement learning, executive functioning, working memory, and neuropsychiatric disorders such as alcohol addiction and pathological gambling [40]. The rs686 polymorphism is located in the 3′ untranslated region of this gene, the dominant Allele A of which increases the risk of PD development (*p* = 0.017), with an estimate of OR = ∞, 95% CI [1.36–∞]. This polymorphism leads to allele-specific effects on the differential expression of the *DRD1* gene, while the C allele shows lower activity compared to the T allele, which is due to the fact that this SNP is located in the miR-504 binding region [40].

### 4.2. Analysis of Complex Genotype Associations in PD Patients

The analysis of complex genotype associations in PD patients was carried out in APSampler software designed to analyze composite genetic biomarkers associated with polygenic disease phenotypes. All associated substitutions: rs2097629, rs1611115, rs6265, rs6275, rs12364283, rs686 were included in the analysis.

We were able to identify a total of 4 PD-associated complex genotypes that were assessed using a permutation test. In three of four cases, the rs6275:T substitution genotype was found in the *DRD2* gene, which resulted in about 9-fold increase in the risk of PD development. Furthermore, a rs2097629:G allele of the *DBH* gene was revealed in two of four cases, which resulted in about three-fold increase in the risk of PD development. It is worth noting that the rs6275:C allele of the *DRD2* gene demonstrates obvious protective properties in relation to PD. Thus, the study showed that the *DBH* and *DRD2* genes had the most pronounced effects on the PD development. No obvious correlations were revealed between the rs2097629 substitution of the *DBH* gene and the PD symptoms, however, it may be assumed that there is an increased risk of the disease as a result of a decrease in the enzyme synthesis in combination with other factors. No data are available on the correlation between the PD development and the rs6275 substitution in the *DRD2* gene. 

### 4.3. Association between the Genetic Markers and ICD in PD Patients

The *BDNF* gene encodes a protein that is active in the spinal cord and the brain. Its main function is to regulate the growth, differentiation and functioning of neurons. The dominant Allele A of the rs6265 substitution increases the risk of ICD development (*p* = 5.7 × 10^−6^), with an estimate of OR = 4.49, 95% CI [2.24–9.18]. This substitution is located in Exon 2 of the *BDNF* gene, and leads to the Val66Met amino acid substitution. The Met allele is associated with abnormal intracellular packaging of the BDNF precursor and a decrease in the cell production of mature BDNF [35]. The association between the rs6265 substitution with OCD, attention-deficit/hyperactivity disorder, anxiety disorders, Parkinson’s disease is well-known, and we reasonably conclude that as the substitution is associated with behavioral disorders, it can be assumed that this polymorphism is associated with ICD.

The TT genotype of the rs6275 substitution in this gene increases the risk of ICD development (*p* = 3.8 × 10^−3^), with OR = 3.16, 95% CI [1.40–6.81]. The C allele is dominant, and the T allele is recessive. As it was mentioned before, this substitution is located in Exon 7 of the dopamine D2 receptor encoding gene and the T allele affects the stability of the DRD2 transcript and its translation efficiency [38]. The T allele effect may be expressed in a decrease in the amount of DRD2 on the presynaptic membrane, dopamine accumulation in the synaptic cleft should be expected. The TT genotype is likely to result in excessive activation of the downstream dopamine receptors and an increased response on the dopamine release. The 5′ region of the gene includes a rs12364283 substitution, the recessive Allele A of which is associated with the ICD development (*p* = 7.7 × 10^−3^) with OR = 3.05, 95% CI [1.29–8.04]. There have been reports on correlation between this substitution and the development of behavioral disorders and dependencies [41], which suggests an association with ICD. The Intron 6 of the *DRD2* gene includes a rs1076560 substitution, the dominant Allele A of which demonstrated a correlation with ICD (*p* = 0.024) with OR = 2.30, 95% CI [1.08–4.83]. There have been reports on the correlation between this substitution and the development of alcohol abuse and drug addiction [42].

The *DBH* gene encodes a protein of the same name that is responsible for the conversion of dopamine to norepinephrine. Dopamine being a key neurotransmitter, having impaired balance in PD patients, was of great interest in our study. The 5′ region of the gene includes a rs1611115 substitution, the recessive Allele C of which is associated with the ICD development (*p* = 1.1 × 10^−4^) with OR = 3.51, 95% CI [1.77–7.29]. This substitution significantly regulates the enzyme plasma activity [34]. In this regard, the impaired function of the dopaminergic system increases the risk of ICD development.

The *ACE* gene, located at 17q23.3, encodes the angiotensin conversion enzyme (peptidyl dipeptidase A). This enzyme is responsible for cleavage of some proteins of the renin-angiotensin system, which regulates blood pressure and the fluid and electrolyte balance in the body [43]. The functional polymorphism rs4646994 is present in Intron 16 in the form of insertion (I) and/or deletion (D) of a sequence of Alu repeats with a length of 289 bp (rs4646994). The dominant Allele I is associated with the ICD development (*p* = 0.024) with OR = 2.64, 95% CI [1.12–7.22]. The I/D polymorphism may affect the *ACE* gene expression and/or the ACE function. Angiotensin II is known to activate several signaling pathways, including mitogen-activated protein kinase (MAPK), phosphoinositide-3-kinase (PI3K)/AKT, and cAMP-dependent protein kinase pathways that play a role in regulating cell growth and differentiation, cytoplasmic protein reorganization, and cell cycle regulation [44].

### 4.4. Analysis of Complex Genotype Associations in PD Patients with ICD

The analysis of complex genotype associations in PD patients was carried out in APSampler software designed to analyze composite genetic biomarkers associated with polygenic disease phenotypes. All associated substitutions: rs1611115, rs6265, rs6275, rs12364283, rs1076560, rs4646994 were included in the analysis.

We were able to identify a total of 4 ICD-associated complex genotypes that were assessed using a permutation test. In three of four cases, there is a *BDNF*_rs6265: A allele, which makes a significant contribution to the development of ICD in PD patients receiving long-term dopaminergic therapy. This allele can independently result in a four-fold increase in the risk of ICD development. However, the *BDNF*_rs6265: G allele demonstrates protective properties in respect of ICD development. An *DRD2*_rs1076560: A allele that was observed in two of four cases and was associated with an increased risk of the disease is of interest for complex genotype analysis. The *DBH*_rs1611115:T allele was found in two of four cases, which independently resulted in about four-fold increase in the risk of the disease.

The *BDNF* rs6265 was shown to correlated with the development of OCD, ADHD and behavioral disorders, which confirms a possible association with ICD (19582215). The *DRD2* rs1076560 substitution might be associated with the development of alcohol abuse and drug addiction, which makes it possible to assume a correlation with the development of ICD as an abnormal behavior. The *DBH* rs1611115 polymorphism is significantly associated with cognitive functions, which explains the probable correlation with ICD [45].

### 4.5. Association between the Genetic Markers and ICD in PD Patients

CT and CC substitutions (rs6275) of the *DRD2* gene increase the risk of ICD development in PD patients (*p* = 0.026) with OR = 2.85; 95% CI [1.04–7.81]. The C allele is dominant, and the T allele is recessive.

The comparison of PD + ICD (49) group and PD group (36) as the internal control showed that the rs6275 substitution in the *DRD2* gene suggested a correlation between the CT and CC genotypes and the PD + ICD phenotype (OR = 2.85), i.e., Allele C has a dominant mode of inheritance for the PD + ICD sample. There is an association between the TT genotype and PD + ICD phenotype (OR = 3.16) (recessive mode of inheritance) as evidenced by the comparison of PD + ICD group versus the population control. There is also a significant association between the TT genotype with the recessive mode of inheritance (OR = 9.00) as evidenced by the comparison of PD without ICD group versus the population control.

The OR values show that the presence of the TT genotype plays a crucial role in the development of PD without related disorders whereas the development of ICD depends more on the presence of the C allele. The presence of a recessive T allele (TT genotype) was observed when comparing PD patients with the control group. The C or T substitutions lead to changes in RNA splicing, which result in altered proportions of the long and short DRD2 receptor isoforms, respectively. The C allele is often a wild-type allele, which has a positive effect on the stability of the DRD2 transcript and the translation efficiency [38]. Normal activity of the *DRD2* gene in PD patients leads to a more effective response to dopamine therapy. Therefore, it can be assumed that PD itself is not the cause of ICD development, and that ICD symptoms may manifest as a result of the use of dopaminergic therapy.

## 5. Conclusions

In summary, we have shown that variants rs1611115 *DBH*, rs6265 *BDNF*, rs6275 *DRD2* rs12364283 *DRD2*, rs1076560 *DRD2*, rs4646994 *ACE* are associated with an increased ICD risk among PD patients. To the best of our knowledge, this is the first report of clinical genetic testing and identification of risk factors for ICD conducted in patients with PD and ICD in Russia. These results would need to be replicated by further studies with a larger population and other ethnic groups as we recognize that the sample size of this study was small although the statistical power was sufficient for analyses. We also acknowledge that our control population group, taken from a biobank of a healthy screened blood transfusion service was not specifically screened for ICD. This fact is a possible limitation towards the conclusions reached. However, as mentioned previously we used a fully health screened blood donor group where all the donors had passed a rigorous medical examination, and those with family history of neurodegenerative disorders, dementia as well any behavioral or mental health issues were excluded. This would mean that those with family history of PD were excluded and furthermore, exclusion of those with significant mental health issues or behavioral disorders would mean that intrusive ICD would have been likely to have been screened out as well.

Special attention should be drawn to rs6275 *DRD2* gene polymorphism. Our data suggest that this specific polymorphism is associated with a strong clinical genetic risk factor for the development of ICD in PD patients and may therefore enable pharmacogenetic strategies to aid personalized treatment while also enabling possible prophylaxis [46]. This issue is also highly relevant in the view of the increasing frequency of “dopamine agonist phobia” which has been recently reported [47]. These studies also contribute to our better understanding of the role of dopaminergic transmission and signaling in the mesocorticolimbic dopaminergic system and the involvement of other neurotransmitter systems in the mechanisms of ICD development. A possible long-term gain may be that the proposed genetic risk factors for ICD development might be used as a biomarker of neurotransmitter dysfunction based nonmotor subtypes of PD [48], allowing a personalized approach to PD therapy [49,50].

## Figures and Tables

**Table 1:** Demographic and clinical characteristics of the study groups.

Parameters described	PD + ICD, PD1, *n* = 49	PD2, *n* = 36
Mean age, years	65.8 ± 8	70.6 ± 5.9
Number of subjects male	23	18
Number of subjects female	26	18
Education duration, years	15.9 ± 3	15.8 ± 3.6
Duration of the disease, years	6.6 ± 4.94	7.53 ± 4.9
Hoehn and Yahr stage	2.2 ± 0.5	2.5 ± 0.5
UPDRS, total score	33.4 ± 11.9	36.3 ± 12.2
LEDD, mg/day	731.5 ± 454	762.4 ± 342.1
Duration of the use of dopaminergic therapy, years	6.6 ± 4.94	7.53 ± 4.9
Breakdown of the types of dopaminergic therapy	Levodopa + DA (*n* = 13; 26.5%), Levodopa + DA + amantadine (*n* = 13; 26.5%), DA monotherapy (*n* = 7; 14.3%), Levodopa monotherapy (*n* = 4; 8.25%), DA + amantadine (*n* = 4; 8.25%), Levodopa + COMT inhibitor + DA + amantadine (*n* = 3; 6.1%), Levodopa + amantadine (*n* = 2; 4.1%), Levodopa + COMT inhibitor + DA (*n* = 1; 2%), Levodopa + MAO-B inhibitor (*n* = 1; 2%), Levodopa + DA + amantadine + MAO-B inhibitor (*n* = 1; 2%).	Levodopa + DA + amantadine (*n* = 14; 38.9%), Levodopa + DA (*n* = 12; 33.3%), DA + amantadine (*n* = 5; 13.9%), DA monotherapy (*n* = 4; 11.1%), Levodopa + COMT inhibitor + DA + amantadine (*n* = 1; 2.8%).

UPDRS = Unified Parkinson’s disease rating scale; LEDD = levodopa equivalent daily dose; DA = dopamine agonists; COMT inhibitor = catechol-O-methyltransferase inhibitor; MAO-B inhibitor = monoamine Oxidase B inhibitor.

**Table 2:** Summary table of statistical analysis for the group of patients with Parkinson’s disease (PD) without impulsive-compulsive disorder (ICD) (PD2) vs. population control group.

Gene	Substitution		PD2	Control	Chi, *p*	Fi (*p*)	OR	CI95%
*DBH*	rs141116007	II + ID	26	282	1.017	0.294	0.67	0.30–1.64
		DD	10	73	0.313		1.49	0.61–3.36
*BDNF*	rs2049046	AA	7	50	0.847	0.327	1.51	0.53–3.76
		AT + TT	29	312	0.358		0.66	0.27–1.90
*DRD2*	rs1799732	CC	15	18	1.911	0.189	1.83	0.71–4.68
		CD + DD	21	46	0.167		0.55	0.21–1.42
*MAOA*	VNTR	SS + SL	20	129	3.309	0.076	1.89	0.89–4.05
		LL	16	195	0.069		0.53	0.25–1.12
*DRD2*	rs6275	TT	18	34	43.706	2.9 × 10^−8^	9.00	3.97–20.14
		CT + CC	18	306	3.8 × 10^−11^		0.11	0.05–0.25
*DBH*	rs2097629	AA	6	114	5.995	0.016	0.34	0.11–0.86
		AG + GG	30	192	0.014		2.97	1.17–8.97
*BDNF*	rs6265	AA + AG	16	94	8.308	6.7 × 10^−3^	2.83	1.27–6.29
		GG	16	266	3.9 × 10^−^^3^		0.35	0.16–0.79
*DBH*	rs1611115	TT + CT	26	328	11.256	2.8 × 10^−3^	0.27	0.11–0.68
		CC	10	34	7.9 × 10^−4^		3.71	1.46–8.77
*COMT*	rs4680	AA + AG	22	147	3.773	0.063	0.48	0.22–1.11
		GG	14	45	0.052		2.08	0.90–4.65
*DRD2*	rs2283265	TT + CT	34	161	0.572	0.610	0.53	0.08–5.78
		CC	2	5	0.449		1.89	0.17–12.13
*DRD2*	rs12364283	TT	30	100	6.877	0.012	3.30	1.25–10.19
		CT + CC	6	66	8.7 × 10^−3^		0.30	0.10–0.80
*DRD2*	rs1076560	TT + CT	14	40	3.305	0.095	2.00	0.86–4.53
		CC	22	126	0.069		0.50	0.22–1.16
*SLC6A4*	rs38130034	TT	13	43	2.762	0.140	1.89	0.81–4.28
		CT + CC	23	144	0.097		0.53	0.23–1.24
*ACE*	rs4646994	II + ID	26	228	0.367	0.708	1.27	0.57–3.05
		DD	10	111	0.545		0.79	0.33–1.77
*SLC6A3*	rs27072	CC	24	86	2.634	0.139	1.86	0.83–4.36
		CT + TT	12	80	0.105		0.54	0.23–1.21
*DRD1*	rs686	CC	0	23	5.629	0.017	0.00	0.0000–0.7362
		CT + TT	36	143	0.018		∞	1.3583–∞

VNTR = variable number of tandem repeats; Fi = Fisher’s test criteria; OR = odds ratio; 95% CI = 95% confidence interval.

**Table 3:** Results of analysis of complex genotype associations in PD2 group patients. An increased risk of PD development.

Informative Allelic Pattern	Genotype Carriers		Fi (*p*)	OR	CI95%
	PD2	Control			
*DBH*_rs2097629:G; *DRD1*_rs686:G; *DRD2*_rs12364283:A,A	75.0%	29.7%	6.38 × 10^−7^	7.10	3.11–16.21
*DBH*_rs2097629:G; *DRD2*_rs6275:T,T	44.4%	8.6%	1.42 × 10^−6^	8.51	3.62–20.04
*BDNF*_rs6265:A; *DRD2*_rs6275:T,T	36.4%	4.3%	1.92 × 10^−6^	12.57	4.45–35.49
*DRD2*_rs6275:T,T	50%	12.3%	2.24 × 10^−6^	7.15	3.20–15.97

Fi = Fisher’s test criteria; OR = odds ratio; 95% CI = 95% confidence interval; *p* (Westfall–Young) < 0.001.

**Table 4:** Results of analysis of complex genotype associations in PD2 group patients A decreased risk of PD development.

Informative Allelic Pattern	Genotype Carriers		Fi (*p*)	OR	CI95%
	PD2	Control			
*BDNF*_rs6265:G; DRD2_rs6275:C	45.5%	86.9%	9.51 × 10^−7^	0.13	0.055–0.29
*DRD2*_rs6275:C	50%	87.7%	2.24 × 10^−6^	0.14	0.063–0.31

Fi = Fisher’s test criteria; OR = odds ratio; 95% CI = 95% confidence interval; *p* (Westfall–Young) < 0.001.

**Table 5:** Summary table of statistical analysis for PD patients with ICD (PD1) vs. population control group.

Gene	Substitution		PD1	Control	Chi, *p*	Fi (*p*)	OR	CI95%
*DBH*	rs141116007	II + ID	38	282	0.312	0.580	0.82	0.39–1.81
		DD	12	73	0.576		1.22	0.55–2.53
*BDNF*	rs2049046	AA	11	50	2.335	0.138	1.76	0.76–3.79
		AT + TT	39	312	0.126		0.57	0.26–1.32
*DRD2*	rs1799732	CC	20	18	2.506	0.156	1.89	0.79–4.52
		CD + DD	27	46	0.113		0.53	0.22–1.26
*MAOA*	VNTR	SS + SL	27	129	3.585	0.065	1.77	0.93–3.39
		LL	23	195	0.058		0.56	0.29–1.07
*DRD2*	rs6275	TT	13	34	10.528	3.8 × 10^−3^	3.16	1.40–6.81
		CT + CC	37	306	1.1 × 10^−3^		0.32	0.15–0.72
*DBH*	rs2097629	AA + AG	39	263	2.110	0.199	0.58	0.27–1.36
		GG	11	43	0.146		1.73	0.74–3.76
*BDNF*	rs6265	AA + AG	27	94	23.224	5.7 × 10^−6^	4.49	2.24–9.18
		GG	17	266	1.4 × 10^−6^		0.22	0.11–0.45
*DBH*	rs1611115	TT	14	209	15.644	1.1 × 10^−4^	0.28	0.14–0.56
		CT + CC	36	153	7.6 × 10^−5^		3.51	1.77–7.29
*COMT*	rs4680	AA	10	52	0.446	0.575	0.77	0.32–1.73
		AG + GG	35	140	0.504		1.30	0.58–3.16
*DRD2*	rs2283265	TT	26	118	2.893	0.105	0.56	0.27–1.17
		CT + CC	19	48	0.089		1.80	2.74–25.02
*DRD2*	rs12364283	TT	37	100	7.512	7.7 × 10^−3^	3.05	1.29–8.04
		CT + CC	8	66	6.1 × 10^−3^		0.33	0.12–0.78
*DRD2*	rs1076560	TT + CT	19	40	5.774	0.024	2.30	1.08–4.83
		CC	26	126	0.016		0.43	0.21–0.93
*SLC6A4*	rs38130034	TT	15	43	2.068	0.179	1.67	0.76–3.56
		CT + CC	30	144	0.150		0.60	0.28–1.31
*ACE*	rs4646994	II + ID	38	228	5.513	0.024	2.64	1.12–7.22
		DD	7	111	0.019		0.38	0.14–0.90
*SLC6A3*	rs27072	CC + CT	43	149	1.452	0.377	2.45	0.55–22.65
		TT	2	17	0.228		0.41	0.04–1.83
*DRD1*	rs686	CC + CT	39	137	0.438	0.653	1.38	0.51–4.34
		TT	6	29	0.508		0.73	0.23–1.96

VNTR = variable number of tandem repeats; Fi = Fisher’s test criteria; OR = odds ratio; 95% CI = 95% confidence interval.

**Table 6:** The result of analysis of complex genotypes in patients with ICD. An increased risk of ICD development.

Informative Allelic Pattern	Genotype Carriers		Fi (*p*)	OR	CI95%
	PD1	Control			
*ACE*_rs4646994:I; *BDNF*_rs6265:A; *DRD2*_rs1076560:A	25.6%	0.006%	2.68 × 10^−7^	55.17	6.80–447.57
*BDNF*_rs6265:A; *DRD2*_rs1076560:A	28.2%	2.5%	3.28 × 10^−6^	15.42	4.58–51.86
*BDNF*_rs6265:A; *DBH*_rs1611115:T	43.2%	12.4%	1.89 × 10^−5^	5.36	2.51–11.44
*BDNF*_rs6265:G; *DBH*_rs1611115:T	72.7%	37.3%	2.63 × 10^−5^	4.49	2.15–9.37

Fi = Fisher’s test criteria; OR = odds ratio; 95% CI = 95% confidence interval; *p* (Westfall–Young) < 0.001.

**Table 7:** The result of analysis of complex genotypes in patients with ICD A decreased risk of ICD development.

Informative Allelic Pattern	Genotype Carriers		Fi (*p*)	OR	CI95%
	PD1	Control			
*BDNF*_rs6265:G; *DBH*_rs1611115:C,C	22.3%	60.8%	5.73 × 10^−6^	0.19	0.09–0.41
*BDNF*_rs6265:G,G; *DRD2*_rs6275:C	27.3%	64.6%	9.77 × 10^−6^	0.21	0.10–0.43

Fi = Fisher’s test criteria; OR = odds ratio; 95% CI = 95% confidence interval; *p* (Westfall–Young) < 0.001.

## Data Availability

The data presented in this study are available on request from the corresponding author. The data are not publicly available due to country specific and ethical committee regulations.

## Supplementary Materials

The following are available online at https://www.mdpi.com/article/10.3390/jpm11121321/s1, Table S1: characteristics of primers and PCR conditions, Table S2: characteristics of primers and probes, PCR—real-time conditions, Table S3: restrictases and restriction fragments.
