# Supplementary material for: Genetic Markers as Risk Factors for the Development of Impulsive-Compulsive Behaviors in Patients with Parkinson’s Disease Receiving Dopaminergic Therapy
Source: J Pers Med. 2021 Dec 7;11(12):1321. doi: 10.3390/jpm11121321 (PMC8706187; doi:10.3390/jpm11121321)
Supplement: Supplementary file 1 [file jpm-11-01321-s001.zip › jpm-1447043-supplementary.pdf]

Supplementary Material.

Genetic markers as risk factors for the development of impulsive-compulsive behaviors in patients with Parkinson's disease receiving dopaminergic therapy

Anna Fedosova[1], Nataliya Titova[2,3,*], Zarema Kokaeva[1], Natalia Shipilova[2,3], Elena Katunina[2,3], Eugene Klimov[1†]

[1]Lomonosov Moscow State University, Faculty of Biology, Leninskie gory, 1, building 12, 119234 Moscow, Russia;

[2] Pirogov Russian National Research Medical University, Moscow, Russia; Ostrovitianova, 1, 117997, Moscow, Russia;
[3]Federal State Budgetary Institution "Federal center of brain research and neurotechnologies" of the Federal Medical Biological Agency, Moscow, Russia; Ostrovitianova, 1, building 10, 117997, Moscow, Russia;
† Deceased on July, 8, 2021;

* Author to whom correspondence should be addressed – Nataliya Titova (e-mail: nattitova@yandex.ru, tel. +79032428792)

Table S1. Characteristics of primers and PCR conditions.

Gene / SNP	Primers 5'-3'	$T_{annealing}$
BDNF<br>rs2049046	F = CAAAGTGTGACTTCAGATTGTCTG<br>R = AGAATAAGACAGCAGTACCGTACTT	56°C
BDNF<br>rs6265	F = GAGGACAAGGTGGCTTGGCCTA<br>R = GGCCGAACTTTCTGGTCCTC	61°C
DBH<br>rs1611115	F = CTAGTCCAGCTGGAGAGATCT<br>R = TTTGCCATCATCCACCCGTG	61°C
DBH<br>rs141116007	F = AATCAGGCACATGCACCTCC<br>R = GGCCCTGAGGAATCTTACAGG	56°C
DBH<br>rs2097629	F = GGCTTGGTGTGGTTAGGATGA<br>R = CCAGGGTCTTGTGCCTCACA	60°C
DRD2<br>rs6275	F = ATGGAGATGCTCTCCAGCAC<br>R = ACCTTTCACAGACCGGGCTG	60°C
DRD2<br>rs1799732	F = AGGACCCAGCCTGCAATCAC<br>R = TGCCGGTTCGGCACTGAAG	60°C

MAOA VNTR	F = ACAGCCTCGCCGTGGAGAAG<br>R = GAACGGACGCTCCATTCGGA	55°C
ACE<br>rs4646994	F = CTCCCATTTCTCTAGACCTG<br>R = GCTCACCTCTGCTTGTAAG	55°C
SLC6A3<br>rs27072	F = CCACGCATCGGGAAAGGACT<br>R = GCACCTCGCCGTGTCTTGT	59°C
DRD1<br>rs686	F = GTGTGTTGGAAAGCAGCAGAG<br>R = CTGTCCCCAGCCCTATCAG	58°C

Table S2. Characteristics of primers and probes, PCR-RealTime conditions. * - SNaPshot

Gene / SNP	Primers 5'-3'	$T_{annealing}$
DRD2<br>rs2283265 *	G = GGAAACAGGCTCATAGAAGGTATGC<br>T = CCGGCGCGGCCGCCGGAAACAGGCTCATAGAAGGTACGA<br>F = TTTTGCTGAGTGACCTTAGGCAA	56°C
DRD2<br>rs12364283 *	A = CTGTCCTCAGTTTGCCGGA<br>G = GCCCGGCGCGCGCCCTGTCCTCAGTTTGCCTGG<br>R = CAGCACCTGTTTAAGCCTCAGT	57°C
DRD2<br>rs1076560 *	C = TTGCAGGAGTCTTCAGAGTGG<br>A = CGGGCCGGCCGCCCGGTTGCAGGAGTCTTCAGAGCGT<br>F = CTGCACCAGAGGCAGAGG	58°C
COMT<br>rs6267	F = GCAGCGCATCCTGAACC<br>R = CTTCTGCTCGCAGTAGGTGTC<br>FAM-CGGGAACGCACAGAGC-BHQ1<br>VIC-CGGGAACTCACAGAGCG-BHQ1	55°C
SLC6A4<br>rs38130034	F = AATAATAACCTCCATACACAATTGAG<br>R = TTCTTATCTCTAGTCCAATATTTTGG<br>FAM-TAGAATTTGTTAATGTAAGAAAAA-BHQ1<br>VIC-GAATTTGTTACTGTAAGAAAA-BHQ	55°C

Table S3. Restrictases and restriction fragments.

Gene	rsID	Endonuclease	Sizes of fragments
BDNF	rs2049046	HinfI	A=163+35; T=198
BDNF	rs6265	PspCI	A=157; G=116+41
DBH	rs1611115	FauI	C=89+77, T=166
DBH	rs2097629	BstMAI	C=179+50, T=132+50+47
DRD2	rs6275	BssT1I	C=343; T=247+96
DRD2	rs1799732	AgsI	C=273; (-)=148+125
SLC6A3	rs27072	MspI	CC=210+145, TT=355

DRD1	rs686	BstC8I	GG=161+71; AA=230
